# Supplementary figures and images for: The Interplay Between Stiffness and Hyperglycemia on Diabetic Foot Ulcer Wound Closure
Source: Cell Mol Bioeng. 2026 Jan 5;19(1):29–42. doi: 10.1007/s12195-025-00877-8 (PMC13031594; doi:10.1007/s12195-025-00877-8)

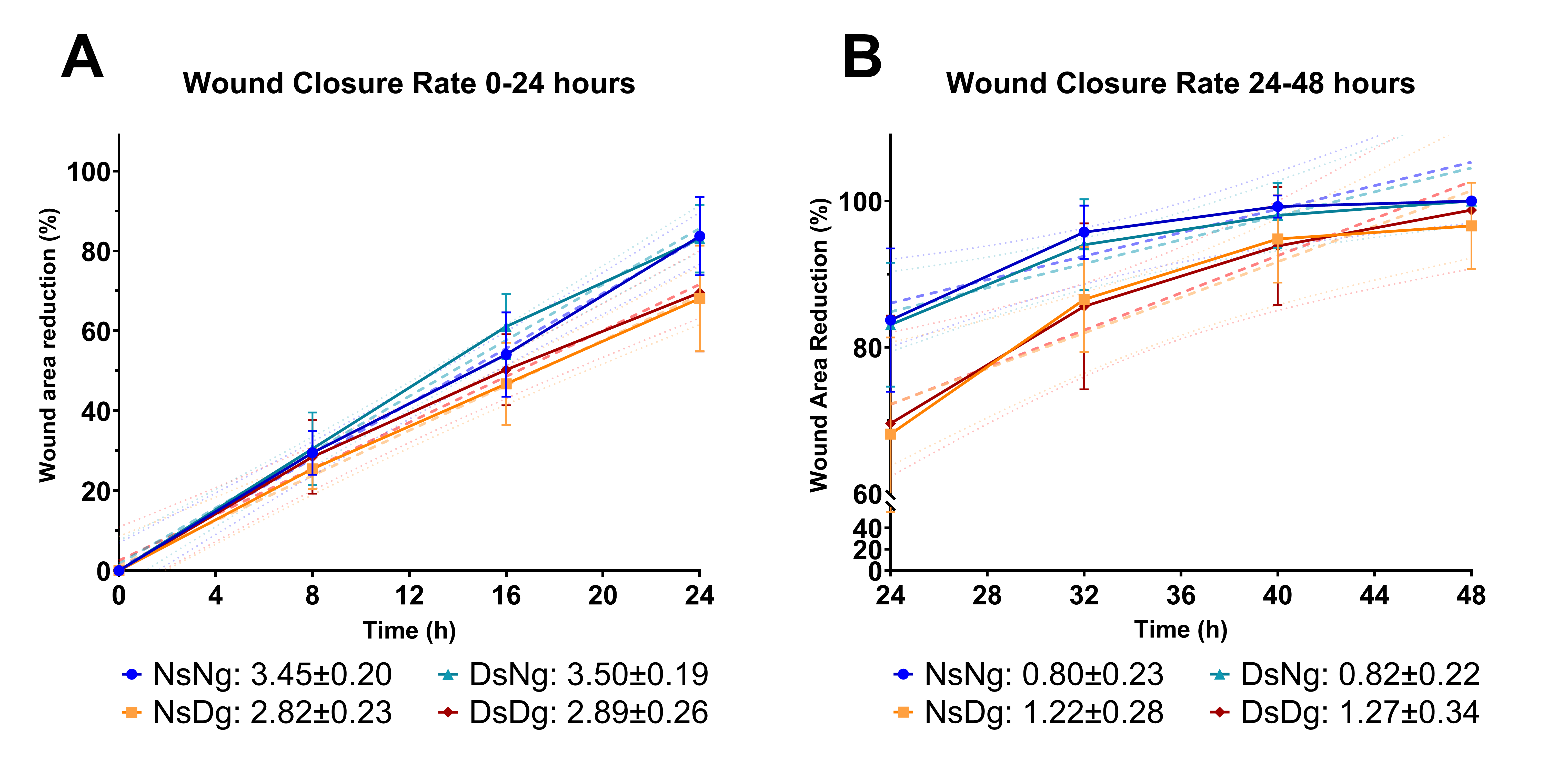

Supplement: Supplementary file 10 — Supplemental Fig. 3 (TIF 1719 kb) [file 12195_2025_877_MOESM10_ESM.tif]
