# Supplementary material for: The Interplay Between Stiffness and Hyperglycemia on Diabetic Foot Ulcer Wound Closure
Source: Cell Mol Bioeng. 2026 Jan 5;19(1):29–42. doi: 10.1007/s12195-025-00877-8 (PMC13031594; doi:10.1007/s12195-025-00877-8)
Supplement: Supplementary file 12 — Supplementary Information (DOCX 28 kb) [file 12195_2025_877_MOESM12_ESM.docx]

The interplay between stiffness and hyperglycemia on diabetic foot ulcer wound closure

Nourhan Albeltagy^1^, Jennifer Patten^1^, Karin Wang^1*^

^1^ Department of Bioengineering, Temple University, Philadelphia, PA 19122, USA.

*Corresponding Author:

Karin Wang

1947 North 12^th^ Street

Philadelphia, PA 19122

[karin.wang@temple.edu](mailto:karin.wang@temple.edu)

# Supplementary codes:

**Code1:** Rose is adapted from (Wind Rose, MATLAB). Cellrose function generates a polar histogram (rose plot) of cell movement directions and speeds. Inputs include a vector of cells’ migration angle and a vector of cell velocities. Output is a 2D histogram matrix representing the percentage of cells in each direction and speed bin. Supplementary include cellrose.m function and cellrose_script.m example to run the function.

**Code2:** Migration tracks script. This MATLAB code imports cell migration data, normalizes and plots individual cell trajectories on a 2D graph, color-coding each track based on its starting quadrant. It also calculates and marks the center of mass of all cell endpoints, providing a visual summary of cell movement patterns over 24 hours. Input is an .xlsx file in the format defined by (ibidi Chemotaxis and Migration Tool, [Chemotaxis and Migration Tool | Free Software | ibidi](https://ibidi.com/chemotaxis-analysis/171-chemotaxis-and-migration-tool.html))

# Supplementary figures:

**Supplemental Fig. 1 Representative wound closure videos.** Wound area reduction was evaluated at time points 0, 8, 16, 24, 32, 40, and 48 hours. Phase images were acquired with a Keyence BZ-X800 fluorescence microscope and a 10x objective. The two substrate stiffnesses are normal stiffness (Ns) at 57±5 kPa and diabetic stiffness (Ds) at 90±6 kPa. The two glucose conditions are normal glucose levels (Ng) at 5.5 mM, and diabetic glucose levels (Dg) at 11.1 mM. A) Wound closure on normal stiffness with normal glucose levels (NsNg). B) Wound closure on normal stiffness with diabetic glucose levels (NsDg). C) Wound closure on diabetic stiffness with normal glucose levels (DsNg). B) Wound closure on diabetic stiffness with diabetic glucose levels (DsDg).

**Supplemental Fig. 2 Representative Trackmate videos.** Trackmate cell tracking was applied to the 48-hour time-lapse acquired with 10-minute intervals, from SPY-595 stained nuclei. Phase images were taken by a Keyence BZ-X800 fluorescence microscope with a 10x objective and a BZ-X Filter TexasRed filter. The two substrate stiffnesses are normal stiffness (Ns) at 57±5 kPa and diabetic stiffness (Ds) at 90±6 kPa. The two glucose conditions are normal glucose levels (Ng) at 5.5 mM, and diabetic glucose levels (Dg) at 11.1 mM. A) Trackmate video of cell nuclei migrating on normal stiffness with normal glucose levels (NsNg). B) Trackmate video of cell nuclei migrating on normal stiffness with diabetic glucose levels (NsDg). C) Trackmate video of cell nuclei migrating on diabetic stiffness with normal glucose levels (DsNg). D) Trackmate video of cell nuclei migrating on diabetic stiffness with diabetic glucose levels (DsDg).

**Supplemental Fig. 3 Wound closure rate between the first and second 24 hours.** A) The wound closure rate in the first 24 hours. The closure rates of normal glucose (Ng) with both normal plantar skin stiffness (NsNg) and higher diabetic plantar skin stiffness (DsNg) are similar, as the slopes do not overlap. The same case within the hyperglycemic glucose levels (NsDg) and (DsDg). The Ng rates increased faster than the Dg and reached 80% of wound closure in the first 24 hours. Slopes with standard error (SE) were: NsNg = 3.45±0.20 (R2=0.95), NsDg = 2.82±0.23 (R2=0.92), DsNg = 3.50±0.19 (R2=0.95), and DsDg = 2.89±0.26 (R2=0.90). B) The second 24 hours is the 24-48 hours range. In this period, the wound closure rate of the NsNg and DsNg conditions slowed due to cell density increase within the wound area. While the NsDg and DsDg conditions still had a high wound closure rate until they reached 80% closure, a decrease in the wound closure rate occurred. The delay in reaching 80% closure, at time point 28 hours, from the Dg conditions caused the wound closure rate to appear faster in the 24-48 hour range. Slopes with standard error (SE) were: NsNg = 0.80±0.23 (R2=0.53), NsDg = 1.22±0.28 (R2=0.60), DsNg = 0.82±0.22 (R2=0.50) and DsDg = 1.27±0.34 (R2=0.53). The low R2 values are caused by the wound closure rate reaching a plateau as wounds are near closure, 80-100%.

**Supplemental Fig. 4 Cell collision guidance in migrating cells closing the wound video.** The video shows an example of a cell migrating to close the wound; it changes direction while migrating under the effect of colliding with other migrating cells, a behavior termed “Cell collision guidance.” Cell1, pointed by the white arrow, is shown where it started migrating at the edge of the wound at time 0, and then reached the first collision with cell2, pointed by the yellow arrow. Cell1 shifted its migrating track slightly to the right. Following, cell1 collided with cell3 and changed the migration direction to the left. And finally, cell1 collided with cell4 and changed its migration direction downwards. Cell1 formed a transient cell-to-cell connection during each of the collisions. The transient connection was lost after a directional change in cell2 and cell4, but was maintained for a short period after collision in cell3. Most of the cells that started near the wound edge had a relatively higher velocity and migrated into the wound area in the first 10 hours. The cells in the back of the crowded area were obstructed by the density of the neighbors and were migrating more slowly. Scale bar = 200 μm. The example wound is under the effect of hyperglycemia and diabetic plantar skin stiffness (DsDg).
